# Supplementary material for: Understanding Attentional Functioning in Adult Attention Deficit Hyperactivity Disorder—Could This Improve Diagnostic Specificity?
Source: Int J Environ Res Public Health. 2023 Mar 14;20(6):5077. doi: 10.3390/ijerph20065077 (PMC10049217; doi:10.3390/ijerph20065077)
Supplement: Supplementary file 1 [file ijerph-20-05077-s001.zip › ijerph-2217446-supplementary.pdf]

Table S1. Questions and prompts

Research Question	Prompts
What are the characteristic experiences of adults with ADHD?	What happens with your attention when you try to study? Or work? Or fill in a form? What is your mind doing?
What models best conceptualise these experiences	We need a model to illustrate ADHD - If your brain was a device, what sort of device do you think it might be? Can you think of an analogy for ADHD?

Table S2. Thematic analysis

**Overarching Theme**	**Theme**	**Subtheme**	**Description (Subtheme)**
Attention	Concentration	Sustained Attention	Continuous attention towards a task/stimulus
		Duration	Average duration of sustained attention on non-leisure tasks
	Distractibility and Flitting Attention	Attention Jumping between Stimuli	Attention jumping from one stimulus/event to another
		Attention Jumping Between Tasks	Jumping from task to task
	Hyperfocusing		Extended and intense fixation on one subject/stimulus
	Multitasking	Multitasking as a Coping Strategy	Multitasking to improve sustained attention and task performance
		Maladaptive Multitasking	Multitasking involuntarily or due to distraction, reducing task performance (e.g., watching videos while working)
	Need for Stimulation		Stimulation seeking behaviour
	Partial Attention		Doing a task without complete focus
Executive Functioning	Decision Making		Proficiency/difficulty with making decisions
	Planning & Organisation		Proficiency/difficulty with planning and organizing tasks/activities
	Prioritising		Proficiency/difficulty with prioritising
	Other Executive Functioning		E.g., inhibition, set shifting
	Overactive Thought Processes		Over-complicating tasks, over-thinking, and racing thoughts
	Procrastination		
Functional Impairment			E.g., social, study-related, occupational, independence
Memory			Memory impairment (of any nature)

Mental State	Boredom/Interest		Boredom or interest towards a task
	Exhaustion/Wakefulness		State of fatigue or wakefulness
	Negative	Stress & Frustration	State of stress or frustration
		Emotional Outbursts	Emotional outburst
	Positive	Motivation	Task related motivation
		Enjoyment	Task related enjoyment
Model	Model: ADHD Medication		Model describing impact of medication
	Model: Distractibility		Model describing distractibility
	Model: Information Use	Computer Model	Model comparing ADHD to a malfunctioning computer
	Model: Hyperfocus		Model describing hyperfocusing
	Model: Intermittent Attention		Model describing intermittent attention
	Model: Meandering Attention		Model describing meandering attention
Task-Related Functioning	Completing Task		Seeing tasks through to completion
	Efficiency		Efficiency of task-related performance
	Information Overload		Overwhelmed by amount of sensory information presented for processing
	Starting Task	Procrastination	Ability to start a task or puts tasks off
